# Serial Detection of Circulating Mucorales DNA in Invasive Mucormycosis: A Retrospective Multicenter Evaluation

**DOI:** 10.3390/jof5040113

**Published:** 2019-12-03

**Authors:** Toine Mercier, Marijke Reynders, Kurt Beuselinck, Ellen Guldentops, Johan Maertens, Katrien Lagrou

**Affiliations:** 1 Department of Microbiology, Immunology and Transplantation, Katholieke Universiteit Leuven, 3000 Leuven, Belgium; 2 Department of Hematology, University Hospitals Leuven, 3000 Leuven, Belgium; 3 Department of Laboratory Medicine, Medical Microbiology, AZ St Jan Bruges, 8000 Bruges, Belgium; 4 Department of Laboratory Medicine and National Reference Centre for Mycosis, University Hospitals Leuven, 3000 Leuven, Belgium

**Keywords:** mucormycosis, Mucorales, circulating DNA, PCR, diagnosis

## Abstract

Invasive mucormycosis is a fungal infection with high mortality. Early diagnosis and initiation of appropriate treatment is essential to improve survival. However, current diagnostic tools suffer from low sensitivity, leading to delayed or missed diagnoses. Recently, several PCR assays for the detection of Mucorales DNA have been developed. We retrospectively assessed the diagnostic and kinetic properties of a commercial Mucorales PCR assay (MucorGenius^®^, PathoNostics) on serial blood samples from patients with culture-positive invasive mucormycosis and found an overall sensitivity of 75%. Importantly, a positive test preceded a positive culture result by up to 81 days (median eight days, inter-quartile range 1.75–16.25). After initiation of appropriate therapy, the average levels of circulating DNA decreased after one week and stabilized after two weeks. In conclusion, detection of circulating Mucorales DNA appears to be a good, fast diagnostic test that often precedes the final diagnosis by several days to weeks. This test could be especially useful in cases in which sampling for culture or histopathology is not feasible.

## 1. Introduction

Invasive mucormycosis, an infection caused by fungi of the Mucorales order, carries a high morbidity and mortality rate. The disease mainly affects patients with uncontrolled diabetes, with extensive trauma of the soft tissues, with hematologic malignancy, or following transplantation [1]. The incidence of this disease appears to be increasing over time, which is likely due to the increasing numbers of patients at risk due to malignancy and uncontrolled diabetes [2,3]. Early and aggressive treatment with systemic antifungals (lipid-based amphotericin B formulations are recommended as first-line agents) and surgery whenever possible is quintessential to improve survival [4,5]. The initiation of adequate antifungal treatment within five days of diagnosis increased the survival threefold compared to treatment that was started after six days of diagnosis (51% versus 17%) [6]. However, the clinical and radiological similarities with invasive aspergillosis, which usually requires a different first-line antifungal treatment, and the documented co-occurrence of *Aspergillus* spp. and Mucorales in up to 45% of cases of mucormycosis further complicate the management of these infections [2]. Indeed, even when a diagnosis of invasive aspergillosis is made, mucormycosis cannot be ruled out with certainty.

Contrary to some other invasive fungal infections, few tools are currently available for diagnosing mucormycosis, leading to frequent and significant diagnostic delay. For example, beta-D-glucan, which is often described as a “pan-fungal” marker, is only produced by Mucorales at levels that are undetectable in serum by current assays [7]. No other antigen-based tests are commercially available to allow fast diagnosis of mucormycosis. However, a seemingly true “pan-fungal” serum disaccharide was recently identified and was positive in 50%–90% of patients with mucormycosis, as well as in 51%–62% of patients with invasive candidiasis and 64%–83% of patients with invasive aspergillosis [8,9]. Unfortunately, this test was only positive before other mycological evidence was obtained in less than one third of the patients with mucormycosis; all other positive serum samples were obtained after a mycological diagnosis had already been made [8,9], making this test more useful as a confirmatory test than as a screening assay. Although these results are encouraging, this test still needs independent validation. Furthermore, Sato et al. identified a *Rhizopus* specific antigen, but this has yet to be evaluated in human mucormycosis and only detects one of the possible Mucorales pathogens [10]. As such, the current diagnosis of mucormycosis continues to rely on traditional microbiologic techniques such as fungal culture, direct microscopy (using optical brighteners), and histology, all of which have a notoriously low sensitivity [11]. This low sensitivity in turn leads to a large number of missed cases, with only one in four cases being diagnosed ante-mortem in a large autopsy series [12].

Recently, molecular techniques have offered an additional opportunity to improve the diagnostic sensitivity [13]. PCR assays using either primers for specific Mucorales genera, as well as pan-fungal assays targeting ribosomal targets followed by ITS sequencing, have successfully been used to identify Mucorales in different matrices, such as bronchoalveolar lavage fluid or tissue specimens [13]. Additionally, two different assays for detecting circulating Mucorales DNA in peripheral blood have been developed [14,15]. The assay by Millon et al. uses three different PCR reactions for the detection of *Mucor/Rhizopus*, *Lichtheimia*, and *Rhizomucor*, respectively [15]. On the other hand, the assay by Springer et al. uses a more generic approach and targets specific fragments of the 18S and 28S genes [14]. The amplicons of the 18S gene need to be sequenced for further identification of the genus. In contrast with other tests, both PCR assays were able to detect mucormycosis before the diagnosis was made by more traditional methods such as histopathology or culture [16,17]. In patients with a positive blood PCR, the molecular test preceded the conventional tests in 80%–90% of patients and was positive up to 250 days earlier. This makes these tests attractive as screening assays, as well as a diagnostic test in cases in which more invasive sampling is not feasible. However, although clinically evaluated [16,18], both tests are “in-house” developed assays and, hence, are not widely available. 

Recently, a commercial semi-quantitative PCR (qPCR) assay was developed (MucorGenius^®^, PathoNostics, Maastricht, The Netherlands). This assay uses a real-time PCR technique to detect DNA from *Rhizopus* spp., *Mucor* spp., *Lichtheimia* spp., *Cunninghamella* spp., and *Rhizomucor* spp. by targeting the 28S rRNA gene in a similar fashion to the assay by Springer et al., and an M13 bacteriophage as internal control. The exact primer sequences are kept confidential by the manufacturer. Recently, the Fungal PCR Initiative (FPCRI) and the ModiMucor study group compared the performance of these assays using spiked serum in a ring test, and found the MucorGenius^®^ and Millon assays to have the highest positivity rates with the lowest Cq values [19]. 

In this retrospective multicenter study, we evaluated the sensitivity of this novel assay for the detection of circulating Mucorales DNA in patients with invasive mucormycosis.

## 2. Materials and Methods 

### 2.1. Data and Sample Collection

We performed a retrospective search of biobanks of stored (at −20 °C) residual fractions of blood samples at the University Hospitals Leuven (Leuven, Belgium) and the AZ St Jan Hospital (Bruges, Belgium). The hospital-based prospective database was queried for positive cultures for Mucorales between 2009 and 2019. The date on which this positive sample was collected was defined as the reference date or “diagnostic” date (D+0). The electronic medical files of the patients with these positive cultures were reviewed for the presence of invasive mucormycosis. Cases were classified according to the 2008 revised EORTC/MSG consensus definitions [20]. We added a classification of “putative” mucormycosis for patients with well-recognized risk factors for mucormycosis (such as diabetic ketoacidosis or iron chelation therapy), but not fulfilling the EORTC/MSG-defined host criteria. For each patient, we collected age, date of diagnostic sample, antifungal therapy, underlying disease, site of infection, time and cause of death, and identification of the Mucorales. We determined the date of start of adequate mucormycosis therapy, defined as liposomal amphotericin B ≥ 5 mg/kg, posaconazole or isavuconazole, as well as the date of the first EORTC/MSG defined clinical signs (i.e., radiological features or endoscopic findings). We retrieved all blood samples (including whole blood, serum, or plasma) from our biobank from two weeks before up to two weeks after the diagnostic date, with a maximum of two samples per week. In case the earliest and/or latest samples in this time window were positive by qPCR, we extended our search period until the samples became negative, or until there were no more stored samples available in the biobank.

This study was approved by the Ethics Committee Research UZ/KU Leuven (study number S62865). The need for informed consent was waived due to the retrospective nature of the study. All data were analyzed using R version 3.6.1 (R Foundation for Statistical Computing, Vienna, Austria). Fisher’s exact test was used to compare the rate of positivity between different groups.

### 2.2. DNA Extraction and qPCR Assay

DNA was extracted from 1 mL of the blood, plasma, or serum sample using the NucliSens easyMAG/eMAG (BioMérieux, France) according to the manufacturer’s instructions. Briefly, samples were pretreated and lysed in a GuSCN-buffer, followed by binding of the free nucleic acid to silica coated magnetic beads. The beads were then washed, after which the bound nucleic acid was eluted into a final volume of 50 µL. The MucorGenius^®^ assay (PN-700, PathoNostics, Maastricht, The Netherlands) was then run using 5 µL of the extracted DNA on a LightCycler 480II (Roche, Basel, Switzerland). This assay contains a premix of Mucorales specific primers for real-time PCR as well as an internal control for quality assurance (yellow and red fluorescent probes respectively). The cycle threshold (Cq) was determined using the fit point method. Any Cq > 40 was set to a fixed Cq of 40. Any detectable amount of DNA (i.e., Cq < 40) was considered a positive result. Positive and negative controls were included in every batch. The turnaround time of the test including DNA extraction was around 3.5 h.

## 3. Results

**Figure 1:**
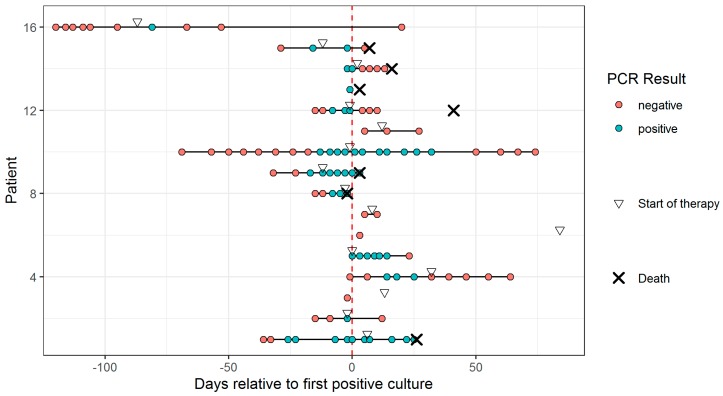
Temporal overview of all tested samples of each patient relative to the time of the first positive culture (Day 0). A cross denotes death of a patient. A triangle indicates start of appropriate Mucorales therapy.

**Figure 2:**
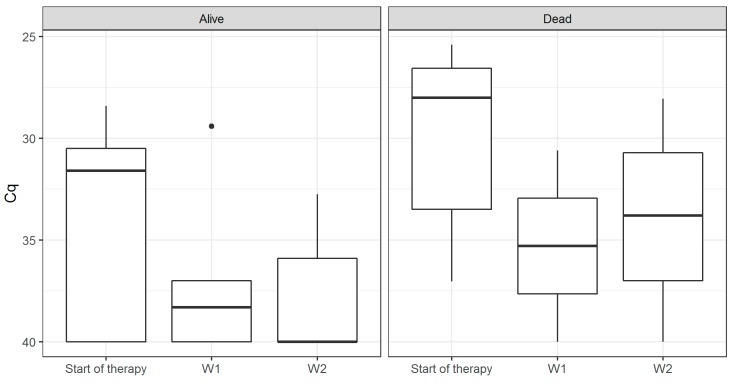
Boxplots of the Cq values of the semi-quantitative qPCR at initiation of treatment, and after one and two weeks relative to the initiation of adequate anti-fungal therapy. The middle line denotes the median, and the box defines the interquartile range. Survival is given at Week 6.

We analyzed 106 blood samples from 16 patients (median 5.5 samples per patient, interquartile range 2.75–9.25). Patient characteristics are shown in Table 1. The overall sensitivity of the assay in peripheral blood was 0.75 (95% CI 0.48–0.93). The rate of positive results appeared to be higher in hematology patients than in non-hematology patients (0.89 vs. 0.57), although this difference was not significant (*p* = 0.262), likely due to the small overall sample size. Similarly, we found a higher sensitivity in patients with proven mucormycosis compared to probable mucormycosis (0.86 vs. 0.67, *p* = 0.585). The temporal evolution of each patient relative to the time of the first positive culture is shown in Figure 1. We could identify a positive result in patients up to 81 days (median eight days, inter-quartile range [IQR] 1.75–16.25) before the first positive culture result, and up to 24 days (median three days, IQR -0.25 to 8.5) before the first signs of fungal infection on imaging. The evolution of the quantitative result relative to the initiation of adequate antifungal therapy is shown in Figure 2. All six patients who died by Week 6 had a positive blood sample, whereas this was only the case in six out of the ten who survived (*p* = 0.234). On average, the Cq values in patients who survived by Week 6 declined by 22% by Week 1, compared to 9% in those who had died (*p* = 0.53). At Week 2, the average decline was 15% of the baseline value in survivors, compared to 8% in those who died (*p* = 0.75). In patients who had a negative qPCR blood test after initiation of appropriate therapy, survival was 75% at Weeks 6 and 12 (*n* = 8). All patients who did not achieve a negative qPCR test died before Week 6 (*n* = 2, *p* = 0.133). All patients who succumbed before Week 6 died of mucormycosis. Autopsy reports in the four patients in whom this was performed showed disseminated disease in all five cases, also involving organs that showed no clear signs of infection pre-mortem such as the liver and spleen. 

## 4. Discussion

In this study, we tested blood samples from patients with culture-positive probable and proven invasive mucormycosis for circulating Mucorales DNA using the commercial MucorGenius^®^ qPCR assay. Mucorales DNA was a sensitive biomarker for invasive mucormycosis, often preceding the final microbiologic diagnosis by several days to weeks. Although sensitivity appeared to be lower in non-hematology patients, this difference was not significant, likely due to the small sample size of this study. Although a positive qPCR result appeared to be a predictor for Week 6 mortality, we could not identify a correlation between declining DNA levels and survival, although the mortality appeared lower in patients who achieved a negative qPCR result after initiation of therapy. Indeed, in the study by Millon et al., survival was significantly higher in patients that became qPCR negative after treatment (48% vs. 4%, *p* < 0.001) [18].

The commercial MucorGenius^®^ assay used in the present study differs significantly from previously published PCR assays for the detection of Mucorales DNA in blood. The assay by Millon et al., which was used in most studies [15,17,18], uses three separate reactions for the detection of four pathogens, whereas the MucorGenius^®^ test uses only a single reaction. The MucorGenius assay therefore requires less reagent, but, conversely, cannot differentiate among the five genera. The assay by Springer et al. [14] can be used in a single reaction as well while amplifying the 18S gene alone; if further identification is warranted, the amplicon can then be sequenced, offering a compromise between the two other assays.

One specific advantage of the MucorGenius^®^ assay is that it can be run in parallel with an *Aspergillus* specific assay by the same manufacturer (AsperGenius^®^). This could be useful for testing bronchoalveolar fluid samples for both molds simultaneously in a single run using four different detection channels (green, yellow, orange, and red for AsperGenius^®^, and yellow and red for MucorGenius^®^). This could be very relevant clinically as we found 8/16 patients to have co-infection with *Aspergillus*. However, this combined approach does not seem to be useful for detection of both molds in blood samples due to the lower sensitivity of *Aspergillus* PCR in blood [21]. The sensitivity of detecting circulating Mucorales DNA appears to be significantly higher than what is usually seen with detection of circulating *Aspergillus* DNA, likely due to the increased fungal burden, which is around 10–100 times higher in mucormycosis [18].

Clinical suspicion of mucormycosis often preceded the final microbiologic diagnosis. This can be inferred for example from the day of initiation of Mucorales-targeted therapy, which often preceded the first positive culture result. A sensitive biomarker from peripheral blood could help confirm the clinical suspicion in these cases.

In one patient, we could detect circulating Mucorales DNA up to 81 days before a final identification of the causal mold was made, despite disappearance of circulating DNA shortly after the initial positive qPCR result. This patient was treated empirically with high-dose liposomal amphotericin B for suspected mucormycosis but was too thrombocytopenic and immune-compromised to undergo surgery. Three months later, the diagnosis was finally confirmed by more conventional techniques when the lower lung lobe, diaphragm, and liver lobe were surgically removed. This is in line with the results from the assays by Millon et al. (earliest 49 days before positive culture, median nine days) and Springer et al. (earliest 250 days before positive culture, median six days) [15,16]. However, these results should be interpreted with caution as they are influenced by the frequency of blood sampling and storage in each study.

We noticed a high incidence of disseminated mucormycosis at autopsy, including in organs that were seemingly not affected ante-mortem. This highlights the importance of blood sampling, which can diagnose mucormycosis without the need for targeted sampling, as not all affected organs are easily identified or easily sampled for further testing. However, the high rate of disseminated disease at autopsy could of course also reflect the high mortality of disseminated disease, which would lead to an overestimation of the true incidence of this disease entity.

Our study has several limitations. As this is a retrospective study, we have to rely on samples that were stored in sufficient quantity for analysis by qPCR. As such, the time to positivity between a positive culture result or changes in imaging is greatly influenced by the frequency of blood sampling prior to these events. For example, when a patient has a clinical syndrome compatible with mucormycosis, but no blood sample was stored, we could not verify if circulating Mucorales DNA was present. Furthermore, the low incidence of mucormycosis led to a small sample size in this study, despite the 10-year study period, making statistical analysis more difficult. All blood samples in our study were serum samples, with the exception of five whole blood samples. Since results from a comparative study on *Aspergillus* PCR suggest that sensitivity may be higher in plasma than in serum, it is possible that our study underestimated the performance of the Mucorales qPCR [22]. Finally, our study was restricted to culture positive patients only. It is therefore possible that the performance of this assay is different in patients where no positive culture can be obtained. However, as we currently lack other diagnostic tools besides histopathology, microscopy, and culture, this could not be avoided easily.

In conclusion, we report a good sensitivity of circulating Mucorales DNA as tested by the MucorGenius^®^ assay for the diagnosis of invasive mucormycosis. This assay allows fast, non-invasive diagnosis, often preceding clinical signs or diagnosis by conventional tests by several days. Based on our results, a large study including a realistic population of cases and controls is required to further investigate the diagnostic potential of this technique.

## Figures and Tables

**Table 1:** Patient population.

Patient	Sex, Age (Years)	Underlying Disease	Localization	Classification	Identification in Culture	Aspergillus Co-Infection	Survival at Week 6	Survival at Week 12	Start of Appropriate Therapy, Antifungal
1	M, 57	ALL	Disseminated (cutaneous, lung)	Proven	*Rhizopus microsporus*	Yes (Culture *A. fumigatus*/*A. terreus*. BDG > 500 pg/mL. Serum GM 6.0)	Dead	Dead	D+6, L-AmB 10 mg/kg
2	M, 8	AML	Disseminated (cerebral, lung)	Probable	*Rhizomucor pusillus*	No	Alive	Alive	D-2, L-AmB 5 mg/kg
3	F, 34	Lung transplant	Lung	Probable	*Mucor species*	No	Alive	Alive	D+13, L-AmB 5 mg/kg
4	M, 29	Lung transplant	Lung	Probable	*Rhizopus species*	Yes (Culture *A. fumigatus*)	Alive	Alive	D+32, posaconazole
5	F, 54	Crohn’s disease	Sinus	Probable	*Lichtheimia species*	No	Alive	Alive	D+0, L-AmB 5 mg/kg
6	F, 50	COPD	Lung	Proven	*Rhizopus rhizopodiformis*	Yes (Culture *Aspergillus* spp., BAL GM 0.5)	Alive	Alive	D+84, isavuconazole
7	M, 54	Diabetic ketoacidosis	Lung	Putative	*Rhizopus microsporus*	Yes (Culture *A. fumigatus*, BAL GM 5.0)	Alive	Alive	D+8, L-AmB 5 mg/kg
8	M, 61	ALL	Disseminated (pleura, pericardium, lungs, myocardium, spleen)	Proven	*Rhizomucor pusillus*	Yes (Culture *A. fumigatus*, BAL GM 2.2)	Dead	Dead	D-3, L-AmB 5 mg/kg
9	M, 58	AML	Disseminated (lung, liver)	Proven	*Lichtheimia species*	Yes (Culture and PCR *A. fumigatus*, BAL GM 5.1, Serum GM 3.9)	Dead	Dead	D-12, L-AmB 5 mg/kg
10	M, 54	Lung transplant	Lung	Probable	*Rhizopus species*	No	Alive	Alive	D-1, posaconazole
11	M, 78	MDS	Lung	Putative	*Rhizopus species*	Yes (Culture *A. fumigatus*, BAL GM 5.6)	Alive	Alive	D+12, L-AmB 10 mg/kg
12	M, 66	Allogeneic SCT	Lung	Probable	*Rhizomucor pusillus*	No	Alive	Alive	D-1, L-AmB 5 mg/kg
13	F, 63	Solid tumor	Lung	Probable	*Lichtheimia species*	Yes (Culture *A. fumigatus*, BAL GM 5.1, Serum GM 0.7)	Dead	Dead	None
14	F, 63	Aplastic anemia	Disseminated (lung, spleen)	Proven	*Rhizopus species*	No	Dead	Dead	D+2, ABLC 10 mg/kg
15	F, 63	AML	Lung	Proven	*Rhizopus species*	No	Dead	Dead	D-12, posaconazole
16	F, 64	AML	Disseminated (lung, liver, diaphragm)	Proven	*Rhizopus microsporus*	No	Alive	Alive	D-87, L-AmB 5 mg/kg

D+0, Day on which the sample was taken which resulted in growth of Mucorales in culture. ABLC, Amphotericin B Lipid Complex; ALL, Acute Lymphoblastic Leukemia; AML, Acute Myeloid Leukemia; BAL, Bronchoalveolar Lavage; BDG, beta-D-glucan; COPD, Chronic Obstructive Pulmonary Disease; F, Female; GM, Galactomannan; L-AmB, Liposomal Amphotericin B; M, Male; MDS, Myelodysplastic Syndrome; SCT, Stem Cell Transplantation.
